# Regulation of the Actin Cytoskeleton by an Interaction of IQGAP Related Protein GAPA with Filamin and Cortexillin I

**DOI:** 10.1371/journal.pone.0015440

**Published:** 2010-11-10

**Authors:** Subhanjan Mondal, Bhagyashri Burgute, Daniela Rieger, Rolf Müller, Francisco Rivero, Jan Faix, Michael Schleicher, Angelika A. Noegel

**Affiliations:** 1 Medical Faculty, Institute of Biochemistry I, Center for Molecular Medicine Cologne (CMMC), Cologne Excellence Cluster on Cellular Stress Responses in Aging-Associated Diseases (CECAD), University of Cologne, Köln, Germany; 2 Institute of Anatomy and Cell Biology and Center for Integrated Protein Science (CIPSM), Ludwig-Maximilians-University, München, Germany; 3 Department of Biological Sciences, The Hull York Medical School, University of Hull, Hull, United Kingdom; 4 Institute for Biophysical Chemistry, Hannover Medical School, Hannover, Germany; Duke University Medical Center, United States of America

## Abstract

Filamin and Cortexillin are F-actin crosslinking proteins in *Dictyostelium discoideum* allowing actin filaments to form three-dimensional networks. GAPA, an IQGAP related protein, is required for cytokinesis and localizes to the cleavage furrow during cytokinesis. Here we describe a novel interaction with Filamin which is required for cytokinesis and regulation of the F-actin content. The interaction occurs through the actin binding domain of Filamin and the GRD domain of GAPA. A similar interaction takes place with Cortexillin I. We further report that Filamin associates with Rac1a implying that filamin might act as a scaffold for small GTPases. Filamin and activated Rac associate with GAPA to regulate actin remodelling. Overexpression of filamin and GAPA in the various strains suggests that GAPA regulates the actin cytoskeleton through interaction with Filamin and that it controls cytokinesis through association with Filamin and Cortexillin.

## Introduction

The elastic and viscous nature of a cell is largely a result of intracellular gel-like cytoskeletal polymers. Actin filaments control cortical plasticity like cytoskeleton-propelled deformations and protrusions, cell motility and cytokinesis. F-actin crosslinking proteins stabilize the three-dimensional network or densely packed bundles of actin filaments. F-actin crosslinking proteins need two F-actin binding sites (ABD) in order to connect neighbouring actin filaments. These can be supplied in a single polypeptide chain (ABP34 and fimbrin) or by dimerization as in Filamin, Cortexillin, α-actinin. The spatial arrangement of the two ABDs along with the length and flexibility of the spacer elements determines whether a crosslinking protein induces bundling or network formation [1]. The signaling cascades regulating the activity of these crosslinking proteins are however not completely understood [2].

Based on the presence of an elaborate cytoskeleton the social amoeba *Dictyostelium discoideum* has been successfully used to study cytoskeleton based processes. Among the F-actin crosslinking proteins that have been identified in *D. discoideum* [3] a number of Calponin homology (CH) domain containing proteins are present. Here two CH domains (CH1 and CH2) form the actin binding domain (ABD) as in α-actinin, filamin and Cortexillin, whereas in fimbrin the actin binding domain is formed by four fimbrin-type CH domains (CHf1-CHf4) [4]. Actin crosslinking proteins with different actin binding sites are ABP34, elongation factor 1α (ABP50), villin-related proteins and dynacortin [5]–[10].

Filamin crosslinks actin filaments, promotes orthogonal branching and plays an important role in maintaining the cortical actin network [11]. *Dictyostelium* Filamin has an N-terminal ABD followed by a rod domain which is composed of six repeated domains of antiparallel β-sheets adopting an immunoglobulin fold. Dimerization is mediated through rod repeat 6 [12], [13]. A growing body of evidence mainly from mammalian cells suggests roles for Filamin in intracellular trafficking and signal transduction. Furthermore it has been implicated in several human diseases [11], [14], [15]. Filamin interacts with caveolin-1 which is implicated in caveolae biogenesis, cholesterol transport and endocytic events [16]. Involvement of Filamin in signal transduction is inferred by its interaction with several components of the NF-κβ pathway and with the small GTPases RhoA, Rac, Cdc42 and RalA, and also with regulators and effectors of small GTPases like Trio, FilGAP, PAK1 and ROCK, and β1 integrin [17]–[21]. Over 50 interactors of Filamin have been identified in the mammalian system, but only two interactors of Filamin have been reported for *Dictyostelium* Filamin so far, namely Filamin interacting protein FIP, which in association with Filamin is important for development [22], and RasD. The RasD-Filamin complex functions in phototaxis [23].

Cortexillin I and II are closely related (60% identity at the amino acid level) F-actin crosslinking proteins that are required for cytokinesis [24]. In the Cortexillins a coiled-coil domain essential for dimerization follows the two N-terminal CH domains. Cortexillin I and II differ in their C terminal domains and only Cortexillin I harbors a PIP_2_ binding site [25]. They are distributed in the cell cortex during interphase but localize to the cleavage furrow with the onset of cytokinesis, where they remain until the daughter cells separate [26]. Translocation to the cleavage furrow is controlled by Rac1 and IQGAP-related proteins establishing a direct link between signaling and cytoskeletal components. Cortexillins have an essential role in cytokinesis as ablation of one or both of the Cortexillins results in cytokinesis defects. Interestingly, in Cortexillin I the C-terminus that harbors a putative PIP_2_ binding site is crucial for localization of Cortexillin I to the cleavage furrow and rescues the cytokinesis defect. This domain is also important for the strong actin bundling activity of Cortexillin I which can be inhibited by PIP_2_ [25].

IQGAP-related proteins constitute a conserved family of scaffolding proteins interacting with cytoskeletal and signaling proteins [27]. They contain a conserved RasGAP homology domain (GRD) followed by a RasGAP C-terminal domain (RGCT). GRD domains do not exhibit RasGAP activity but interact with activated Rho GTPases and inhibit their GTPase activity thereby acting as Rho GTPase effector [28]. Mammalian IQGAPs can dimerize and harbour an N-terminal CH domain through which they directly bind to F-actin and crosslink filaments [29], [30]. *D. discoideum* has four IQGAP-related proteins [31]. DGAP1 and GAPA which have been previously studied are involved in cytokinesis [32], [33]. Furthermore DGAP1 regulates the F-actin/G-actin ratio in cells [34]. In contrast to mammalian IQGAPs they do not have a CH domain.

In previous work a quaternary complex containing activated Rac1A, cortexillin I and II and either DGAP1 or GAPA had been described [35]. We report here the direct interaction of Rac1a and cortexillin I with GAPA. We identified Cortexillin I as a GAPA interacting protein and narrowed down the interaction of GAPA and Cortexillin to the GRD domain of GAPA and the ABD domain of cortexillin I. The GRD domain is also responsible for the GAPA-Rac1a interaction.

We show further that GAPA also affects the F-actin/G-actin ratio in a cell and identify its interactions with Filamin and Cortexillin I. The interaction with Filamin takes place between the GRD of GAPA and the F-actin binding domain (ABD) of Filamin and may provide an F-actin binding site to GAPA. Filamin can associate with activated Rac1a implying that Filamin regulation of GAPA is downstream of Rac activation. This interaction is independent of GAPA, indicating that Filamin provides an assembly point for small GTPases in order to coordinate remodelling of the actin filament system.

## Materials and Methods

### Cloning of full length GAPA and GRD domain of GAPA

Full length GAPA was amplified from *D. discoideum* strain AX2 cDNA and cloned into pGEM-TE (Promega), and recloned into pBsrN2 vector [36] for expression in *D. discoideum* cells and pGEX-4T1 (GE Healthcare) for expression in *E. coli* BL21. A fragment encoding amino acid residues 202–557 encompassing the GRD domain was amplified from cDNA and cloned in pGEX-4T1 (GE Healthcare) for expression in *E. coli* BL21.

### Cell culture, cell lines and transformation of *D. discoideum* cells

Cells of *D. discoideum* AX2 wild type strain and of transformants were cultivated in liquid nutrient medium at 21°C on polystyrene plates or in shaking culture at 160 rpm [37]. Cells were transformed by electroporation as described [38].

The following cell lines were used in this study. Wild type AX2 cells, AX2-derived myosin II^−^ (mhcA^−^) strain HS2205 [39], AX2-derived GAPA^−^ strain [35], AX2-derived Filamin deficient (FLN^−^) strain HG1264 [40], AX2-derived Cortexillin I^−^ [24] and cells expressing GAPA fused to green fluorescent protein (GFP) at the N-terminus in the above mentioned strains (this work). The following GFP-tagged Filamin polypeptides expressing HG1264 cells have been used in this study: GFP-ABD [41], containing the actin binding domain (ABD) of Filamin; GFP-ABD+rod1-2 containing the ABD and the first two rod repeats of Filamin; GFP-rod1-6, containing the six rod repeats of Filamin; GFP-FLN, in which GFP was present at the C-terminus of Filamin and GFP-FLN^S174A^, a full length Filamin mutated at position 174 in the ABD [42].

### Oligomerization of GAPA

Analytical gel filtration analysis was done by Sephadex G200 column chromatography using cell lysates containing GFP-GAPA or Sephadex G75 column chromatography for the purified GRD domain using the SMART system (GE Healthcare). Cell lysates were prepared by lysis in a buffer containing 25 mM Tris/HCl, pH 7.5, 150 mM NaCl, 5 mM EDTA, 0.5% Triton-X100, 1 mM DTT, supplemented with protease inhibitors (Sigma). Cell lysates were centrifuged for 100,000 g for 15 min at 4°C prior to gel filtration.

### Protein-protein interaction studies

Interaction between GAPA and Filamin was studied using GST-pulldown assays. Glutathione sepharose beads coated with GST-GAPA or GST-GRD were incubated with lysates from AX2 cells at 4°C to pulldown endogenous Filamin. Pulldown eluates were resolved by SDS-PAGE and immunoblotted with Filamin monoclonal antibody (mAb) 82-454-12 [40]. Glutathione sepharose beads or beads coated with GST were used as a control. To map the domain in Filamin which interacts with GAPA, beads coated with GST-GRD were incubated with lysates from HG1264 cells expressing the actin binding domain (ABD), the ABD along with first two rod repeats (ABD+rod1-2), the six rod repeats (rod1-6) or full length Filamin or a mutated Filamin (S174A) as GFP-fusion proteins. Pulldown eluates were resolved by SDS-PAGE and probed with GFP specific mAb K3-184-2 [43].

Interaction between GAPA and Cortexillin was studied by immunoprecipitation of GFP-GAPA using mAb K3-184-2 from extracts of cells expressing GFP-GAPA. Pulldown eluates were resolved by SDS-PAGE and immunoblotted with polyclonal antibodies against Cortexillin I [35]. Also, glutathione sepharose beads coated with GST-GAPA were incubated with cell lysates to pulldown endogenous Cortexillin I. Pulldown eluates were resolved by SDS-PAGE and immunoblotted with Cortexillin I polyclonal antibodies.

Interaction of activated Rac1a with GAPA was investigated by expressing the GRD as a GST fusion and binding it to glutathione sepharose beads. Beads were then incubated with lysates of cells expressing GFP-GAPA including 10 µM MgCl_2_ and either 100 µM GDP or 100 µM GTPγS in the cold for 3 hrs. Beads were washed extensively and eluates were resolved on SDS-PA gels and probed with GFP specific mAb K3-184-2 and Filamin specific mAb 82-454-12.

For interaction of Rac GTPases with GRD of GAPA, 4×10^7^ cells expressing *Dictyostelium* Rac proteins as GFP fusions were lysed in a buffer (25 mM Tris/HCl, pH 7.5, 150 mM NaCl, 5 mM EDTA, 0.5% Triton X-100, 1 mM NaF, 0.5 mM Na_3_VO_4_, 1 mM DTT) supplemented with protease inhibitors (Sigma) and incubated with equal amounts of GST-GRD bound beads for 3 hrs at 4°C. Beads were extensively washed with wash buffer (25 mM Tris/HCl, pH 7.5, 150 mM NaCl, 5 mM EDTA). The pulldown eluates were immunoblotted and Rac proteins detected using GFP specific monoclonal antibodies.

### Fluorescence microscopy

To observe the localization of GFP-GAPA during cytokinesis, cells were first seeded on glass coverslips and synchronized using nocodazole (10 µM/ml) for 3 hrs to block cell division in mitosis. The block was then released by washing away the drug and allowing the cell cycle to progress for 1 hr. Cells were fixed by ice-cold methanol. For α-Tubulin staining rat mAb YL1/2 [44] was used to identify mitotic cells. DNA was stained with DAPI (4′, 6-diamidino-2-phenylindole, Sigma). Actin was recognized by mAb act-1 [45] in methanol fixed cells or by staining with TRITC phalloidin in paraformaldehyde fixed cells. Analysis was by confocal microscopy (Leica TCS SP5).

### Yeast two-hybrid assay

The GRD domain of GAPA was cloned into the yeast two-hybrid vector pACT2 (BD Biosciences Clontech, Palo Alto, CA). DNA fragments carrying the G12V or equivalent (constitutively active) mutation of human and *Dictyostelium* Rho GTPases were generated from wild-type cDNA by polymerase chain reaction-based site-directed mutagenesis. In all cases, the CAAX motif was either modified by mutagenesis (Cys to Ser) or removed by restriction enzyme digestion. Rho GTPases were cloned into the yeast two-hybrid vectors pGADT7, pAS2-1, or pGBKT7 (BD Biosciences Clontech). For RacA, only the GTPase domain was cloned, and for RacH a truncated protein (residues 1–163) was used because full-length constructs activated the β-galactosidase reporter. Constructs in pGADT7 were introduced into yeast strain Y187. Constructs in pAS2-1 or pGBKT7 were introduced into yeast strain Y190. After mating, interactions were estimated by colony-lift β-galactosidase filter assay.

### Miscellaneous Methods

Relative F-actin content was measured by isolating Triton X-100 insoluble cytoskeletons [46] and staining with TRITC labelled phalloidin. The F-actin content was normalized with respect to total protein content in the respective cell type. mAb 135-409-16 [47] was used to detect cap34 as loading control, glutathione S transferase (GST) was recognized by polyclonal antibodies [48].

## Results

### Localization of GAPA

**Figure 1:**
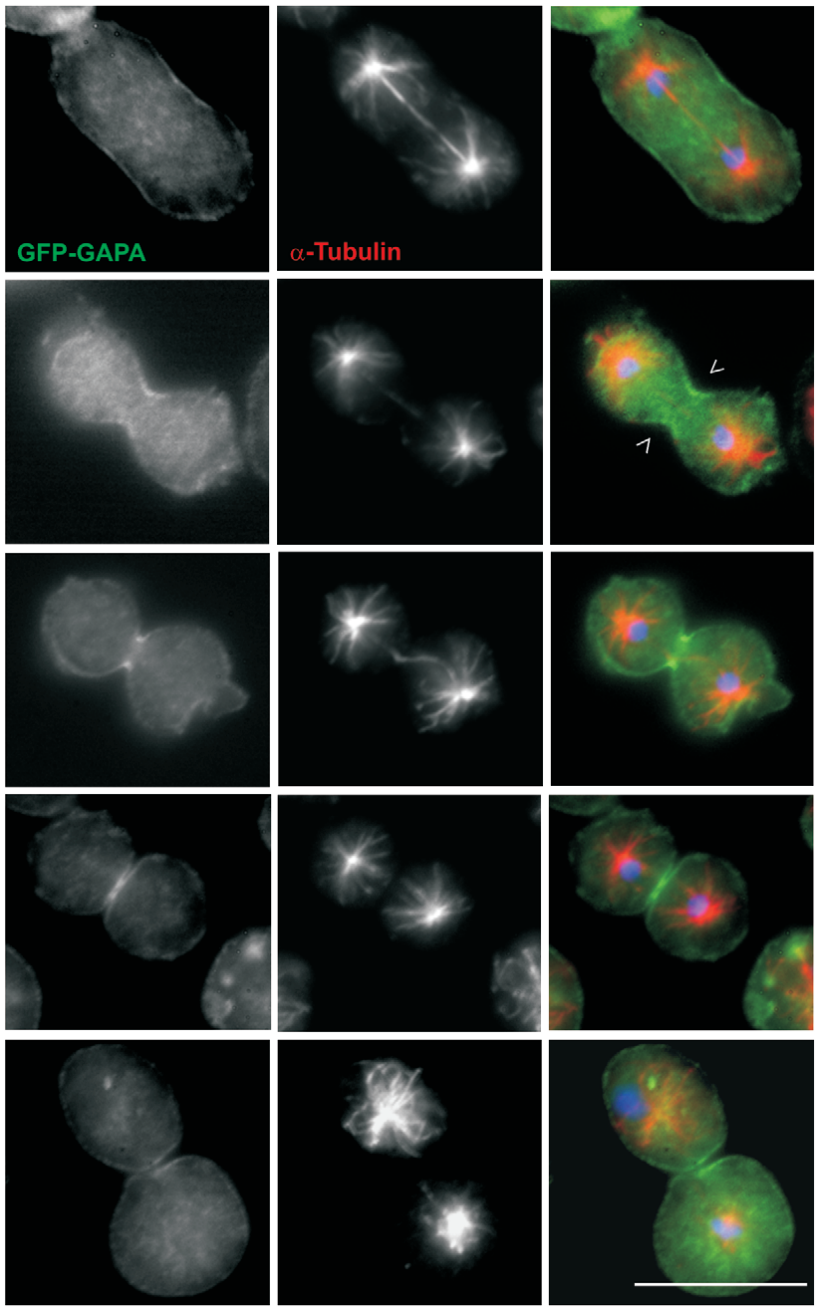
Localization of GAPA during cytokinesis. Localization of GAPA to the cleavage furrow (open arrow heads). Cells expressing GFP-GAPA (green) were synchronized using nocodazole to block progression of the cell cycle and then released and fixed using cold methanol. Tubulin staining (red) is used to identify mitotic cells. Nuclei (blue) are stained with DAPI. Bar, 10 µm.

To analyze the subcellular localization of GAPA we expressed full length GAPA as a GFP fusion in wild type AX2 cells as no GAPA specific antibodies are available. GFP-GAPA is present throughout the cytosol and is strongly enriched at the cell cortex. During cytokinesis it translocated to the cleavage furrow (Figure 1). In GAPA^−^ cells expression of GFP-GAPA completely rescued the strong cytokinesis defect indicating that the fusion protein is functionally active (Figure S1. A). As myosin II is essential for cytokinesis and localizes to the cleavage furrow during cytokinesis, we analyzed the localization of GFP-GAPA in myosin II heavy chain null cells (*mhcA*). *mhcA^−^*cells have a cytokinesis defect when grown in suspension, however when kept on a surface they form a cleavage furrow by an attachment assisted mitotic cleavage and traction mediated cytofission [49]. We found that localization of GFP-GAPA to the cleavage furrow during cytokinesis was unaltered in the *mhcA^−^* mutants (Figure S1. C) pointing out that localization of GAPA and myosin II to the cleavage furrow occurs independently. This is in agreement with results of Adachi et al. (1997) who showed that in GAPA^−^ cells undergoing cytokinesis myosin II is localized in the cleavage furrow. Myosin II independent cytokinesis is mediated by proteins like Cortexillin, Profilin, Talin or Coronin in *Dictyostelium* [50].

### Oligomerization of GAPA

**Figure 2:**
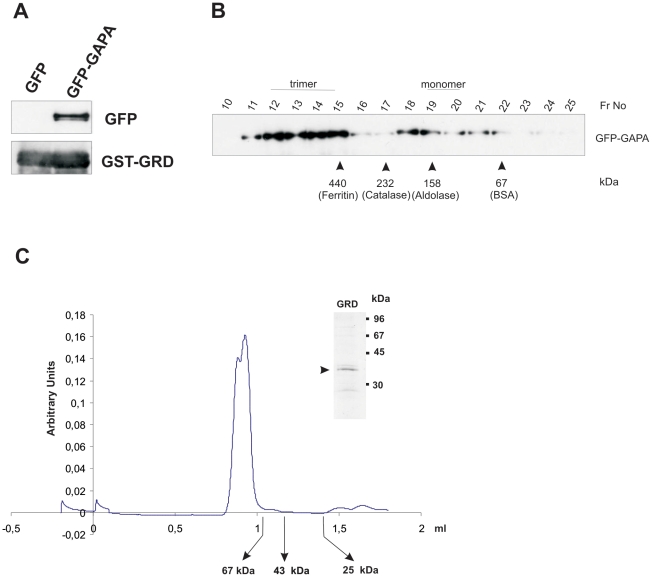
Oligomerization of GAPA. **A.** GST-pulldown showing that GST-GRD pulls down GFP-GAPA from cell lysates. Beads coated with GST-GRD were incubated with cell free extacts from cells expressing GFP-GAPA or GFP. Pulldown eluates were probed with GFP specific antibody mAb K3-184-2. **B.** Cell free extracts from cells expressing GFP-GAPA were subjected to analytical gel filtration. The eluates were analysed by western blotting using mAb K3-184-2. The fraction numbers of elutions of corresponding molecular weight markers are indicated with arrowheads. **C.** Oligomerization of the GRD domain of GAPA using gel filtration chromatography. The GRD polypeptide was subjected to gel filtration chromatography and the OD_280_ recorded to detect the protein. The elution of molecular weight markers is indicated. Inset shows the eluted protein (Coomassie Blue stain).

As mammalian IQGAPs can dimerize we tested this also for the GRD domain of GAPA [29]. GST-GRD could pulldown GFP-GAPA from the soluble extract indicating the ability of GAPA to form oligomers (Figure 2A). When fractionating cell extracts from AX2 cells expressing GFP-GAPA by gel filtration chromatography the 120 kDa GFP-fusion protein largely eluted in fractions over 440 kDa (Figure 2B). We ruled out the possibility that the GFP-tag affected oligomerization by using the 37 kDa recombinant GRD domain of GAPA and performing gel filtration chromatography. In this experiment the GRD domain was largely a trimer (Figure 2C). These results indicate that GAPA can form oligomers, preferably trimers in vivo and that the GRD domain is involved in oligomerization.

### GAPA interacts with Filamin

**Figure 3:**
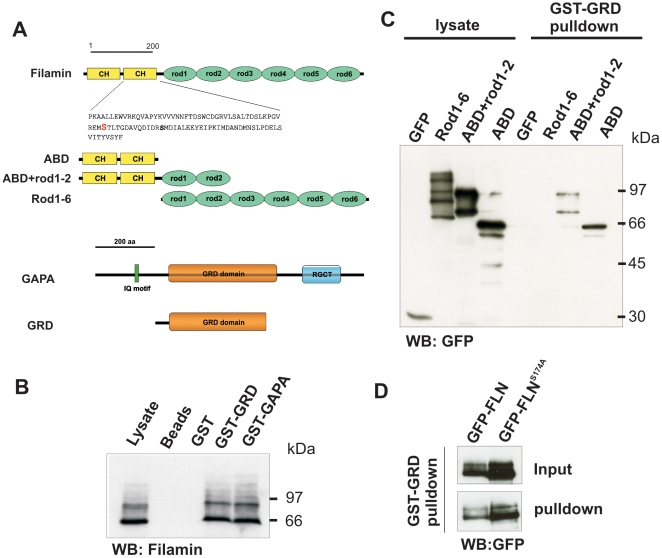
Association of GAPA with Filamin. **A.** Filamin and GAPA polypeptides used in this study. In the upper part the location of a putative phosphorylation site in the CH-domain 2 of Filamin is shown. **B.** Glutathione sepharose beads coated with GST-GAPA or GST-GRD have the ability to pulldown endogenous Filamin detected with mAb 82-454-12 (Brink et al., 1990). The multiple bands observed are due to proteolytic degradation of the protein. **C.** GST-pulldown showing the ability of GST-GRD to bind specifically to GFP-tagged ABD or ABD+rod1-2, but not to rod1-6 constructs of Filamin. **D.** GST-GRD binding to GFP fused full-length Filamin (GFP-FLN) is unaffected in a Filamin mutant (GFP-FLN^S174A^) where a PKA phosphorylatable Serine residue is mutated to Alanine.

GAPA was found as a Filamin interacting protein in immunoprecipitation assays using Filamin monoclonal antibodies followed by MALDI-TOF mass spectrometry. We confirmed the association using different GAPA polypeptides and the Filamin deficient (FLN*^−^*) strain HG1264 expressing various GFP-tagged Filamin polypeptides (Figure 3A). In GST-pulldown experiments both full length GAPA and the GRD domain could pulldown endogenous Filamin from cell lysates (Figure 3B). GST-GRD was then used to map the GAPA interacting part in Filamin. GFP-ABD and GFP-ABD+rod1-2 could bind to GST-GRD but not GFP-rod1-6 harboring the complete rod portion which indicated that the ABD in Filamin acts as the GAPA interacting domain (Figure 3C). A putative PKA phosphorylatable serine residue in the ABD, S174, is however not involved in this interaction as GST-GRD could associate with both GFP-FLN and GFP-FLN^S174A^ where S174 is exchanged to an alanine residue (Figure 3D).

### Filamin overexpression leads to partial rescue of the GAPA*^−^* phenotype

**Figure 4:**
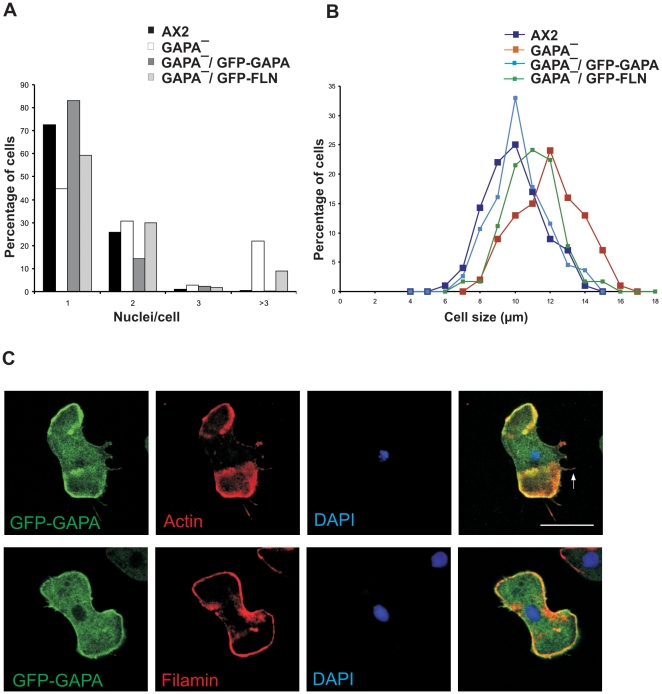
Analysis of cytokinesis and cell size evaluation. **A.** Determination of the nuclei number per cell. The bar graph was obtained by counting the number of nuclei per cell in AX2 and GAPA mutants (at least x number of cells were analysed). **B.** Determination of cell size. Log phase cells (AX2 and GAPA mutants) were harvested and washed twice with Soerensen phosphate buffer (17 mM Na^+^/K^+^ phosphate buffer, pH 6.0) and resuspended at a density of 1×10^7^ cells/ml with the same buffer supplemented with 20 mM EDTA and shaken at 160 rpm at 21°C until cells were rounded. The diameters of spherical cells were measured. At least X number of cells were analysed. **C.** Distribution of GFP-GAPA, actin and Filamin in GAPA^−^ cells. GAPA^−^ cells overexpressing GFP-GAPA were allowed to settle on coverslips coated with poly-l-lysine, methanol fixed, stained with mAb 82-454-12 for Filamin and mAb act1 for actin. Detection was performed with Alexa 568 secondary antibody. Nuclei were stained with DAPI. Bar, 10 µm.

GAPA null cells show a strong cytokinesis defect. They can initiate the formation of the cleavage furrow, but often fail to complete cytokinesis and thus form multinucleated and enlarged cells [32]. Although the activated form of Rac1a recruits IQGAP related proteins into a quaternary complex with cortexillin I and II [35], it is still unknown whether the GAPA-cortexillin interaction itself ensures the completion of cytokinesis. To evaluate the effect of Filamin in the GAPA mutant, we overexpressed full length Filamin in GAPA null cells as a GFP fusion (GFP-FLN) (Figure S1. D). As shown in Figure 4A, 22% of GAPA^−^ cells have more than 3 nuclei. Filamin overexpression partially rescued the cytokinesis defect of GAPA null cells as only 9% of GAPA^−^ GFP-FLN cells had more than 3 nuclei. In addition, it rescued the abnormal and enlarged cell size of GAPA*^−^* cells (Figure 4B). Filamin is an essential component in cytokinesis of dividing chick embryo cells, as it was found highly concentrated in the cleavage furrow, where it remains associated with the midbody region at the completion of cell division [51]. To analyse the localization of filamin in dividing GAPA^−^ cells we perfomed microscopic study using GAPA^−^ GFP-FLN cells treated with nocodazole. Unlike chick embryo filamin, *Dictyostelium* Filamin does not localise at the cleavage furrow but it is highly enriched at the polar region of dividing cells (Figure S1. E). Taken together, it might well be that Filamin facilitates cytokinesis by a so far unknown mechanism.

To analyse and compare the subcellular localization of GAPA and Filamin we performed immunofluorescence studies. We found GFP-GAPA at the cell cortex, where it co-localized with actin, and in the cytosol. GFP-GAPA was also present in actin-labeled filopodia (Figure 4C, arrow). In co-immunofluorescence analysis using Filamin-specific monoclonal antibodies GAPA co-localized with Filamin to a large extent at the cell cortex (Figure 4C).

### GAPA^−^ cells have an altered F-actin content

**Figure 5:**
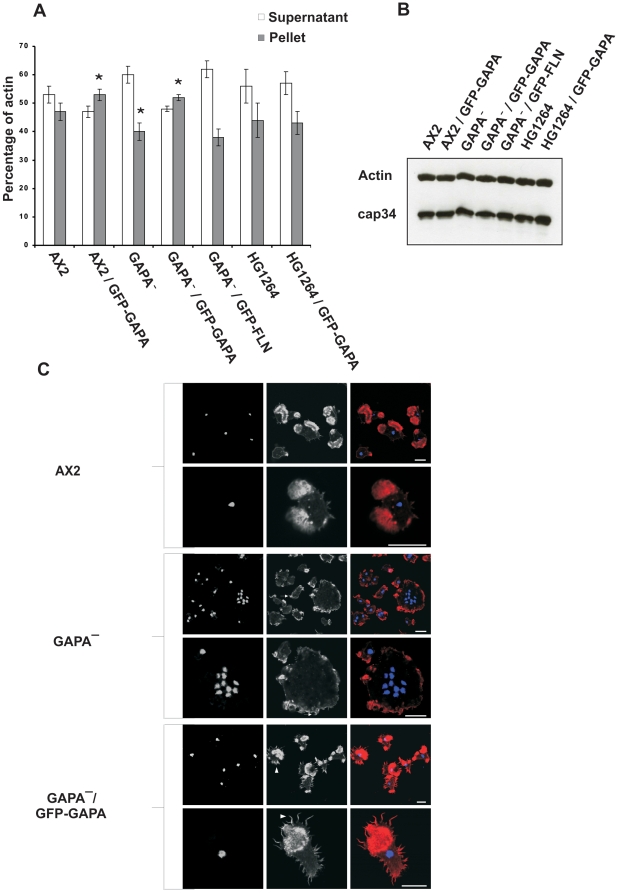
Aberrant F-actin content and its organisation in GAPA and Filamin mutants. **A.** Quantification of F-actin in AX2 wild type, GAPA mutants and Filamin mutants by isolating Triton X-100 soluble material and insoluble cytoskeleton. The cells were lysed in Triton X-100 buffer and the soluble cytoskeleton fraction (supernatant) and the insoluble cytoskeleton fraction were obtained following centrifugation (10 min, 10,000 rpm). The actin content in pellet and supernatant was determined by quantification of the actin bands by Image J software and set in relation to the AX2 values. **B.** The total cellular homognates of AX2 wild-type, AX2 expressing GFP-GAPA, GAPA null mutant, GAPA null mutant expressing GFP-GAPA, GAPA null mutant expressing GFP-FLN, Filamin-deficient HG1264 and HG1264 expresssing GFP-GAPA were loaded per lane, subjected to SDS-PA gel electrophoresis, blotted onto nitrocellulose membrane and labelled with anti actin mAb act-1 and anti cap34 mAb 135-409-16. The actin signals were normalised with respect to cap34 signals in the respective cell types. **C.** Organisation of F-actin. AX2, GAPA^−^ and GAPA^−^ expressing GFP-GAPA cells were fixed with paraformaldehyde and labelled with TRITC-phalloidin to visualise F-actin. Bar, 10 µm. * Significant differences between pellet actin of AX2 and GAPA^−^ cells. *P*<0.05.

The IQGAP related protein DGAP1 affects the organization of F-actin and plays an important role in regulating the G-actin/F-actin equilibrium [34]. To test whether GAPA has a similar effect on the actin cytoskeleton, we prepared Triton insoluble cytoskeletons from AX2, GAPA^−^ and Filamin deficient HG1264 (FLN^−^) cells. Briefly, AX2, GAPA^−^ and FLN^−^ cells were lysed with Triton X-100, and the amount of actin in the pellet and supernatant was quantified by Image J software. Quantification was done by setting the AX2 F-actin content as 100%. GAPA^−^ cells have an approximately 14% lower F-actin content than AX2 cells while AX2 and GAPA^−^ cells overexpressing GAPA have a 12% and 10% higher F-actin content, respectively (Figure 5A). This effect is opposite to that of DGAP1, in which DGAP1 null cells have an increased F-actin content [34] and clearly points towards a role for GAPA in regulating the G-actin/F-actin ratio. Although the F-actin content in the HG1264 strain was reduced, the difference was not significant. Interestingly, overexpression of GAPA in HG1264 did not have an affect on the F-actin content (Figure 5A). Taken together, it appears that GAPA regulates the F-actin content and that Filamin is an essential mediator for this regulation as no effect on the F-actin content was observed when GAPA was overexpressed in HG1264. Furthermore, we also performed western blot analysis on cell lysates of all the mutants to check the total actin content and found that it was unaltered (Figure 5B).

Next we examined the effect on the F-actin content microscopically by performing immunofluorescence analysis using phalloidin staining of F-actin. In AX2 cells F-actin was present at the cell cortex, in filopodia and pseudopodia, while many GAPA null cells showed F-actin rich small projections and very short filopodia (Figure 5C, arrow). GAPA overexpression rescued the aberrant F-actin organization of GAPA null cells. They also formed prominent potrusions and filopodia (Figure 5C, arrow head) and showed an increased F-actin staining when compared to AX2.

### GAPA interacts with Cortexillin I

**Figure 6:**
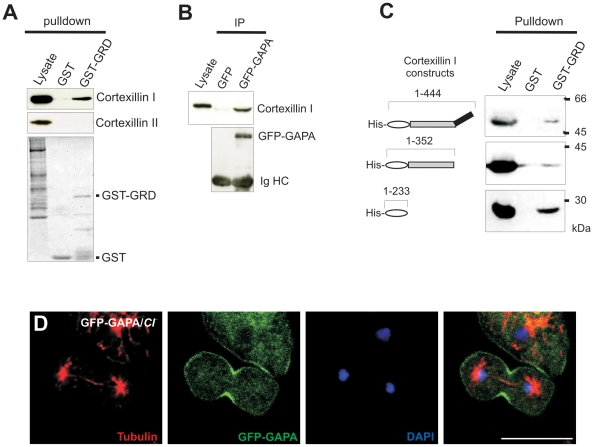
Association between Cortexillin I and GAPA. **A.** Glutathione sepharose beads coated with GST-GRD have the ability to pulldown endogenous Cortexillin I, but not Cortexillin II or another IQGAP related protein DGAP1 (data not shown). **B.** GFP specific mAb K3-184-2 was used to precipitate GFP-GAPA from cells overexpressing GFP-GAPA, the immunoprecipitates were resolved by SDS-PAGE and probed for Cortexillin I. **C.** GST-GRD domain bound to glutathione sepharose beads preferentially interacts with His tagged actin binding domain of cortexillin I. **D.** Localization of GFP-GAPA in the cleavage furrow of Cortexillin I null cells. Cortexillin I null cells expressing GFP-GAPA were synchronized using nocodazole to block progression of the cell cycle and then released, and fixed using cold methanol. Tubulin (red) mAb was used to identify mitotic cells. Nuclei (blue) are stained with DAPI. Images were captured using florescence microscopy. Bar, 10 µm.

Cortexillins are F-actin bundling proteins that organize actin filaments preferentially into antiparallel bundles that then form three-dimensional meshworks [24]. Cortexillins, like GAPA, are enriched in the cortex during interphase and in the cleavage furrow during cell division. To test whether a direct interaction between GAPA and Cortexillin exists, glutathione sepharose beads coated with GST-GRD were used in pull down experiments. GST-GRD could pull down Cortexillin I from cell lysates, but not Cortexillin II (Figure 6A). In immunoprecipitation experiments using cells expressing GFP-GAPA with anti-GFP monoclonal antibody K3-184-2 we could also pull down Cortexillin I, indicating formation of a complex between GAPA and Cortexillin I in vivo (Figure 6B). To further narrow down the interaction site of cortexillin I, we performed pulldown assays using GST-GRD as bait with His-tagged full length cortexillin I, ABD-CC (actin binding domain-coil coiled) domain and ABD domain of cortexillin I. We found that the ABD of cortexillin interacts with the GRD domain of GAPA (Figure 6C). Since both GAPA and Cortexillin are recruited to the cleavage furrow during cytokinesis we investigated whether GAPA localizes to the cleavage furrow in the absence of Cortexillin I. Previous studies had shown Cortexillin I at the cleavage furrow in the absence of either GAPA or DGAP1, but localization to the cleavage furrow was dramatically affected in cells lacking both GAPA and DGAP1 [35]. When we expressed GFP-GAPA in cells lacking Cortexillin I we found that the localization of GAPA to the cleavage furrow during cytokinesis was not affected indicating that localization of GAPA to the cleavage furrow is independent of Cortexillin I (Figure 6D).

### Rac association with GAPA and filamin

**Figure 7:**
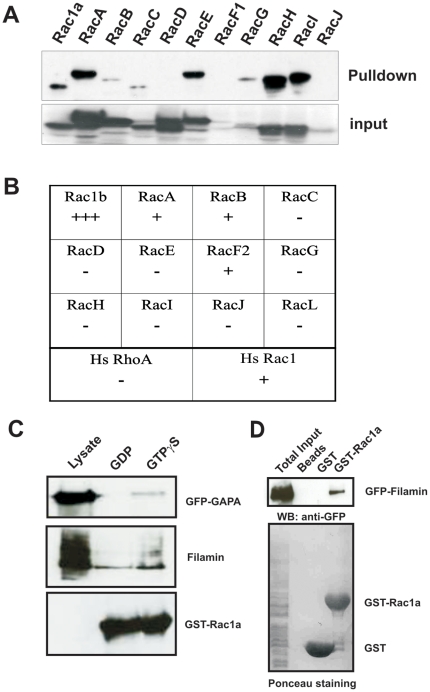
Association of GAPA with activated Rac GTPases. **A.** Interaction of GFP-tagged Rac proteins with GST-GRD in a pulldown experiment. Upper panel (pull down), GFP-fusions pulled down using GST-GRD; lower panel (input) showing whole cell lysates probed for the GFP-fusion proteins. **B.** Interaction of constitutively activated Racs with GRD in a yeast two-hybrid assay represented in a table. **C.** GFP-GAPA associates preferentially with activated Rac1a. Filamin is also present in the complex. GFP-GAPA and Filamin were recognized using appropriate antibodies, GST-Rac1a using GST polyclonal antibodies. **D.** Pulldown of Filamin using GST-Rac1a. Rac1a could pull down GFP-FLN which was recognized by mAb K184-3, GST and GST-Rac1a were visualised by Ponceau S staining.

*D. discoideum* lacks a typical Cdc42 or Rho GTPase, but codes for 18 Rac related small GTPases [31]. We used GST pulldown and yeast-two hybrid methods to analyze Rac GTPases binding to the GRD domain of GAPA. GST-GRD was incubated with AX2 cell lysates expressing different Rac proteins as GFP fusion. The expression levels of the Rac proteins varied strongly. RacF1, G and J were not expressed in high amounts, on the other hand Rac A, B, C, D, E, H, I were highly expressed. We found that, although GST-GRD could pull down Rac1a, A, B, C, E, G, H and I, the interaction was more profound for Rac1a, A, E, H (Figure 7A). We also tested the interaction in yeast two-hybrid assays with constitutively activated forms of Rac to verify the pulldown results. Rac1b showed a strong interaction, RacA, RacB also scored positive followed by RacF2. We then tested mammalian RhoA and Rac1 for association with GAPA and found an interaction for activated Rac1 (Figure 7B). Next we used GST fused Rac1a bound to glutathione sepharose beads and incubated them with lysates of cells expressing GFP-GAPA in the presence of either 100 µM GDP or GTPγS. We found that GFP-GAPA co-sedimented with Rac1a preferentially in lysates that had been treated with GTPγS indicating that the binding is specific for activated Rac1a (Figure 7C).

IQGAPs in general act as Rac effector molecules interacting with activated Rac GTPases. DGAP1 was also shown to preferentially associate with activated Rac1 GTPases [52]. Furthermore, DGAP1, Cortexillin I and activated Rac1a form a complex in vitro [35]. We also detected Filamin in the Rac1a precipitate (Figure 7C). Mammalian filamin was shown to interact with small GTPases, including Rac, Rho, Cdc42 and Ral [20]. We analysed whether such an interaction exists for *Dictyostelium* Filamin as well and performed GST pull down assays with GST fused Rac1a and lysates of cells expressing GFP-FLN in GAPA^−^ (Figure 7D). Western blot analysis revealed that Filamin could interact with Rac1a in the absence of GAPA pointing out that Filamin and Rac1a interaction is independent of GAPA. This indicates that Filamin might act as a scaffolding molecule to bring signaling components near the site of action.

Similarly it has been previously shown that activated Rac could pull down Cortexillin I even in the absence of DGAP1 and from this it was proposed that another protein, namely GAPA, links Rac to Cortexillin [35]. Our findings that activated Rac1a interacts with the GRD domain of GAPA and that GAPA associates with Cortexillin I are in line with the observation of Faix et al. [35] that Cortexillin I forms a complex with either DGAP1 or GAPA in vivo and that these complexes are both required for proper cytokinesis. Furthermore, in a GAPA/DGAP1 double mutant activated Rac does not pull down Cortexillin I [35].

## Discussion

### Association of Filamin with GAPA regulates the F-actin cytoskeleton

We identified GAPA as a Filamin associating protein in *D. discoideum* by immunoprecipitation and GST-pulldown experiments and showed that the ABD of Filamin associates with the GRD domain of GAPA. Purified Filamin constitutively crosslinks actin filaments in vitro, but no clear regulation of this activity has emerged from past studies [53]. The present working hypothesis of the regulation of Filamin's ability to crosslink actin filaments involves its binding partners Rac, Rho and Cdc42 and probably further signaling components that initiate actin filament formation followed by organisation of the filaments into crosslinked actin gels.

For avian gizzard Filamin phosphorylation by CaM-kinase II was shown to prevent its association with F-actin [54], but such mechanisms have not been observed for human or *Dictyostelium* Filamin. Instead, in humans Ca^2+^-bound calmodulin dissociates F-actin from FLNa and inhibits the ability of FLNa to bind and crosslink actin filaments into gels in vitro [55]. IQGAP1 was identified as a calmodulin binding protein that interacts with both F-actin and Cdc42 and has been thought to be a link between Ca^2+^/calmodulin and Cdc42 signaling [56], [57]. GAPA contains a putative IQ motif that can mediate binding to calmodulin as in IQGAP1, allowing that Ca^2+^ affects GAPA functions in actin remodeling. A role of GAPA-Filamin interaction in F-actin reorganization and maintaining an intact cortical cytoskeleton is supported by the findings that cells lacking GAPA have a lower F-actin content, and cells overexpressing GFP-GAPA have higher F-actin levels. Filamin null cells overexpressing GFP GAPA do not show elevated levels of F-actin. In accordance with this we observed a tendency of formation of nuclei free particles in both Filamin and GAPA null cells which presumably arise from a weakened cortical cytoskeleton resulting in membrane blebs (data not shown).

The small GTPases coordinate functions of actin binding proteins at the cell cortex. However, small GTPases require a scaffold on which they can instruct various components in order to achieve their coordinated action. We show that Filamin could function as such a scaffold in *D. discoideum*. In mammalian cells, FLNa interacts with small GTPases and also with factors upstream and downstream of GTPases as a scaffold. These interactions take place at repeat 23 and 24 of FLNa [20], [58], [59]. Mammalian Filamin links the peripheral actin cytoskeleton to transmembrane receptors that are involved in processes such as motility, adhesion or cell shape changes [60], [61]. However, no interactions have been identified between Filamin and membrane receptors or glycoproteins in *D. discoideum* cells. Further investigation will be necessary to determine such interactions.

Our findings suggest that filamin could act as a scaffold for Rac1a-GAPA interaction. GAPA preferentially interacts with the activated form of Rac1a suggesting that GAPA is downstream of Rac1a in signal transduction, whereas the Rac1a-Filamin-GAPA complex could coordinate actin remodeling. Althuogh Filamin null cells do not show a cytokinesis defect, Filamin overexpression rescued the GAPA^−^ phenotype in cytokinesis which depicts that involvement of Filamin in cytokinesis might be independent of GAPA. Interestingly, Filamin overexpression in GAPA^−^ did not affect the F-actin content of GAPA null cells, which points out that the Filamin-GAPA complex is indispensable in actin reorganisation However, Filamin does not localize to the cleavage furrow in GAPA^−^ cells, therefore it is unclear how Filamin regulates cytokinesis in GAPA^−^ cells which will need further investigation.

### Association of Cortexillin I with GAPA in regulation of cytokinesis

We identified GAPA at the cleavage furrow during cytokinesis and found that expression of GFP-GAPA in GAPA null cells rescues the cytokinesis defect. We also identified Cortexillin I as a GAPA interacting protein. Cortexillin I localizes to the cleavage furrow during cytokinesis as does GAPA. Elimination of Cortexillin I and II causes a severe cytokinesis defect. Furthermore, recruitment of Cortexillin I and II to the cleavage furrow is regulated by Rac1a and IQGAP-related proteins GAPA and DGAP1 revealing a network of interactions that converge during cytokinesis. The function of GAPA has been thought to be mediating signal transduction through small GTPases. Loss of RacE leads also to reduction in cortical tension and a strong cytokinesis defect when grown in suspension [62]. GST pulldown experiments showed that GST-GRD can associate with Rac1a and RacE among other Rac GTPases (data not shown).

In mammalian systems Rho GTPases also modulate the mechanical strength of the cortex. Activation of Rho leads to activation of Rho activated kinase and citron kinase which regulate myosin II light chain kinase (MLCK). MLCK phosphorylates myosin II and facilitates myosin II filament assembly in the cleavage furrow. Myosin II is the primary motor protein required for cytokinesis and in *D. discoideum* myosin II null cells Cortexillin I accumulates in form of a compact ring in the cleavage furrow [26]. We found that localization of GAPA was unaffected in myosin II null cells implying that myosin II is not involved in recruiting GAPA to the cleavage furrow.

**Figure 8:**
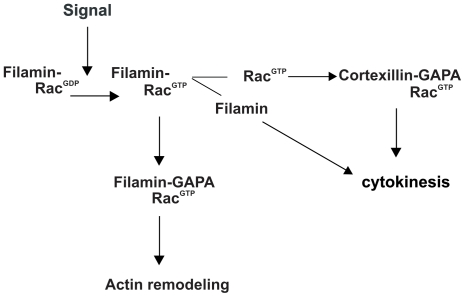
Model representing the role of GAPA and its association with Filamin and Cortexillin I in regulating the actin cytoskeleton and cytokinesis.

Our findings suggest the regulation of cytokinesis through Filamin and an additional mode of actin reorganization through GAPA-Filamin interaction. Current models propose that activated Rac forms a complex with GAPA and cortexillin which then regulates cytokinesis. Here we show that small GTPases can associate with filamin and get activated upon certain signals. Filamin bound activated Rac1a further forms a complex with GAPA thus indicating that GAPA is a downstream effector of Rac activation for actin remodeling. Furthermore the Filamin-Rac-GAPA complex regulates actin remodeling (Figure 8).

## Supporting Information

Figure S1 **A.** Expression of GFP-GAPA rescues the cytokinesis defect in GAPA^−^ cells. Nuclei are stained with DAPI. Bar, 10 µm. **B.** Vegetative wild type cells expressing GFP-GAPA were analysed using confocal microscopy. The Inset shows a fluorescence intensity profile of GFP-GAPA distribution in a representative cell (measured using Image J software) through an arbitrary position in the cell marked by the white line. **C.** Localization of GAPA to the cleavage furrow is independent of myosin II. Cells expressing GFP-GAPA (green) were synchronized using nocodazole to block progression of the cell cycle and then released, and fixed using cold methanol. Tubulin (red) mAb is used to identify mitotic cells. Nuclei (blue) are stained with DAPI. Bar 10 µm. **D.** GFP-FLN expressing GAPA^−^ cells fixed with methanol and nuclei stained with DAPI. Images were taken by confocal microscopy, Bar, 10 µm. Overexpression of Filamin was confirmed by western blot analysis. **E.** Localization of Filamin in cells forming a cleavage furrow. GAPA^−^/GFP-FLN cells were synchronized using nocodazole and then released and fixed with methanol. Tubulin (blue) and cortexillin (red) were recognized by appropriate antibodies. Bar, 10 µm.
