# Supplementary figures and images for: Regulation of the Actin Cytoskeleton by an Interaction of IQGAP Related Protein GAPA with Filamin and Cortexillin I
Source: PLoS One. 2010 Nov 10;5(11):e15440. doi: 10.1371/journal.pone.0015440 (PMC2978108; doi:10.1371/journal.pone.0015440)

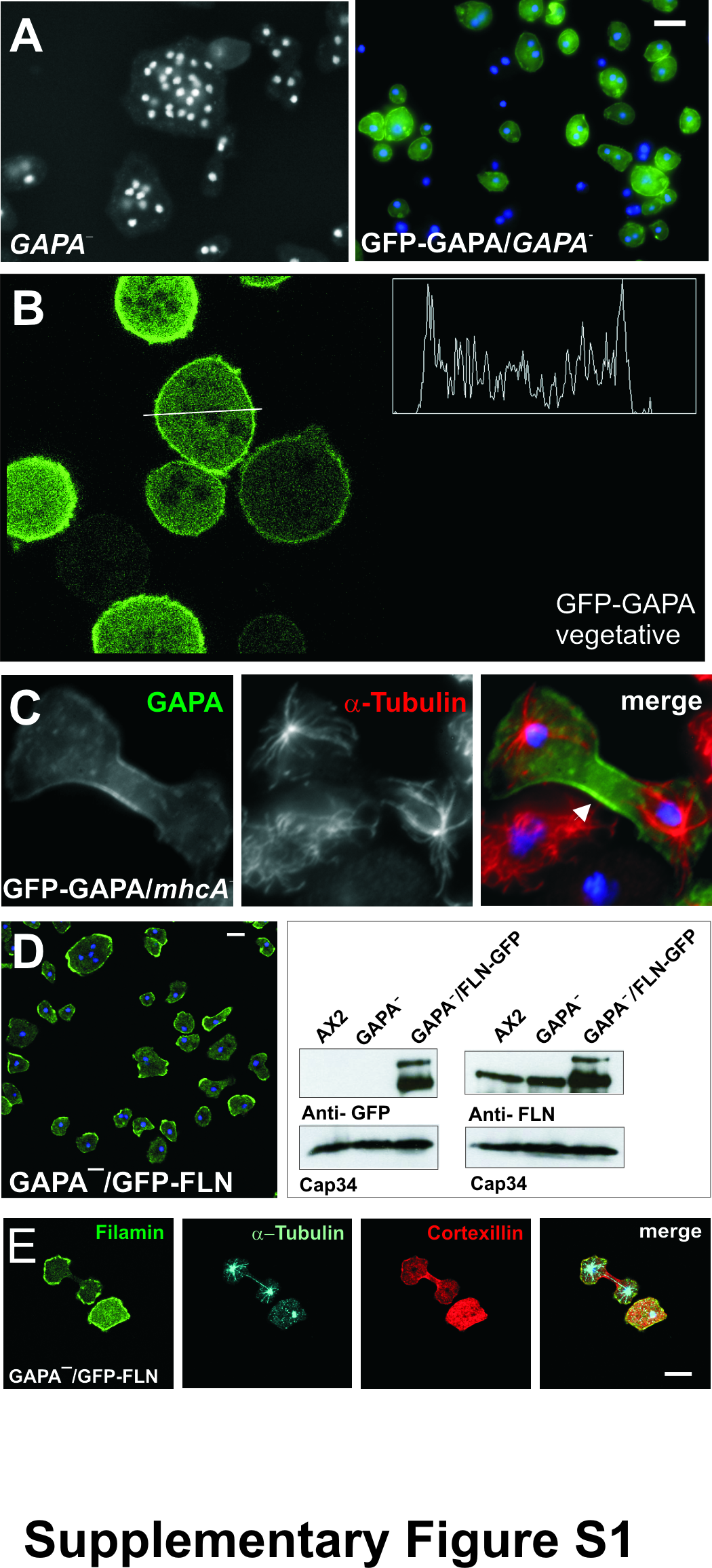

Supplement: Figure S1 — A. Expression of GFP-GAPA rescues the cytokinesis defect in GAPA− cells. Nuclei are stained with DAPI. Bar, 10 µm. B. Vegetative wild type cells expressing GFP-GAPA were analysed using confocal microscopy. The Inset shows a fluorescence intensity profile of GFP-GAPA distribution in a representative cell (measured using Image J software) through an arbitrary position in the cell marked by the white line. C. Localization of GAPA to the cleavage furrow is independent of myosin II. Cells expressing GFP-GAPA (green) were synchronized using nocodazole to block progression of the cell cycle and then released, and fixed using cold methanol. Tubulin (red) mAb is used to identify mitotic cells. Nuclei (blue) are stained with DAPI. Bar 10 µm. D. GFP-FLN expressing GAPA− cells fixed with methanol and nuclei stained with DAPI. Images were taken by confocal microscopy, Bar, 10 µm. Overexpression of Filamin was confirmed by western blot analysis. E. Localization of Filamin in cells forming a cleavage furrow. GAPA−/GFP-FLN cells were synchronized using nocodazole and then released and fixed with methanol. Tubulin (blue) and cortexillin (red) were recognized by appropriate antibodies. Bar, 10 µm. [file pone.0015440.s001.tif]
